# Medical residents’ and teachers’ perceptions of the digital format of nation-wide didactic courses for psychiatry residents in Sweden: a survey-based observational study

**DOI:** 10.1186/s12909-022-03989-1

**Published:** 2023-01-05

**Authors:** Rajna Knez, Samir El Alaoui, Josefin Ivarson, Lise-Lotte Risö Bergerlind, Sarantos Stasinakis, Anna-Maria Ahlgren, Martin Maripuu, Danielle Talaee Mofrad, Klara Bolander Laksov, Nitya Jayaram-Lindström, Karolina Sörman

**Affiliations:** 1 Gillberg Neuropsychiatry Centre, Institute of Neuroscience and Physiology, Sahlgrenska Academy, University of Gothenburg, Kungsgatan 12, 411 19 Gothenburg, Sweden; 2 Department of Clinical Neuroscience, Centre for Psychiatry Research, Karolinska Institute & Stockholm Health Care Services, Stockholm County Council, Norra Stationsgatan 69, 113 64 Stockholm, Sweden; 3 Department of Healthcare, Region Västra Götaland, Regionens Hus, 405 44 Gothenburg, Sweden; 4 Division of Psychiatry, Department of Clinical Sciences, Umeå University, Umeå, Sweden; 5 Department of Education, Stockholm University, Stockholm, Sweden

**Keywords:** Graduate medical education / psychiatry residency, COVID-19 pandemic, Online education, Curriculum development

## Abstract

**Background:**

This study aimed to explore residents’ and teachers’ perceptions of the digital format of Metis (a national education network in Sweden) didactic courses for psychiatry residents in Sweden to guide post-pandemic curriculum development.

**Methods:**

An online attitude survey was developed and sent out to 725 residents in psychiatry and 237 course directors/teachers. Data were examined descriptively and group differences were analysed with independent sample *t*-tests.

**Results:**

The survey was completed by 112 residents and 72 course directors/teachers. Perceptions of digital formats were quite similar between the two groups with some significant differences i.e., residents agreed more strongly than directors/teachers with the statement that Metis courses in digital format were of the same quality (or better) than the classroom-based format. Residents perceived the positive effects of using interactive tools more than directors/teachers. More than 40% of the responders in both groups preferred a return to classroom-based course meetings. Responders in both groups suggested that different forms of digital elements (e.g., video-based and sound-recorded lectures, digital-group discussions, virtual patients) could be incorporated into different phases in the courses.

**Conclusions:**

The study represents the current largest survey among residents in psychiatry and a teaching faculty in Sweden, to understand the impact of digitalization on the quality of residents’ education during the pandemic. The results point towards applying a mixed format for training and education going forward, incorporating digital aspects into the national curriculum.

## Introduction

The COVID-19 pandemic has challenged traditional teaching systems globally, at the same time as it has served as a potential catalyst for creative change, not least within the field of medical education [1, 2]. The rapid digitalization taking place during this time will likely influence the design and format of education and next-generation medical curricula. Articles related to COVID-19 and psychiatry education emphasize several central questions which need to be explored further. For instance, initial findings by Heldt et al. [3] suggested that even though resident psychiatrists and their teachers generally perceived in-person didactic courses as superior to remote learning, the majority wished to retain a digital format to some extent even after the restrictions were phased out. Koraym et al. [4] surveyed how directors of psychiatric residency programmes perceived the shift to digital course formats by highlighting the opportunity to turn the crisis-driven ‘emergency digitalization’ into effective hybrid models to maintain training and education.

The current study aims to explore residents’ and teachers’ perceptions of nationwide didactic courses for psychiatry residents which were rapidly digitalized as a result of the COVID-19 pandemic. The results will offer perspectives from a Swedish setting and contribute to informing the next generation of course recommendations for graduate psychiatric education in a post-pandemic era.

## Methods

Metis is a national educational network in Sweden that provides didactic courses that fulfil the mandatory learning objectives prescribed by the National Board of Health and Welfare for the training of residents in the different disciplines of psychiatry (child and adolescent, general, old age, forensic, and addiction). There are 41 different course concepts that residents may choose from. Given that some courses are held several times per year (offered in different cities), there is a total of 60 courses held nationally each year.

From its inception in 2007, the pedagogical model used by Metis has been in line with the “flipped classroom” concept, mixing distance and in-classroom learning. An overall goal is to provide the participant with practical clinical skills based on sound theoretical knowledge. Courses are offered at different geographical locations in six regions, enabling networking between physicians from different clinics. Such training is aimed at contributing to the establishment of a nationally standardized quality of care.

A Metis course typically consists of three phases: Phase I (a distance-based self-study, which is part-time for four-weeks, comprising designated literature and the completion of mandatory quizzes and written assignments), Phase II (classroom-based course meeting days, typically for three consecutive days, comprising lectures and supervised peer-exercises on clinical cases) and Phase III (examination, distance-based, part-time for four-weeks, usually comprising an examination utilizing the newly obtained knowledge in a written assignment of a clinical case or giving a lecture at the clinic). In this context, distance-based refers to course aspects generally taking place at the resident’s home clinic (or at home).

A few courses were completely digitalized during the Spring of 2020. Between August 2020 and the end of the current study period (i.e., August 2021), practically all Metis courses were offered in a digital format (i.e., course meeting days in Phase II were given in a digital format). At the start of the digitalized period, the Metis central office organized a half-day workshop tailored for course directors, teachers and administrators within the Metis network, to concretely demonstrate and improve skills in the use of digital tools (e.g., interactive discussion sessions referred to as breakoutrooms). An opportunity to exchange experiences of digitalized courses was provided along with access to pedagogical policy documents with interactive elements. Target group policy documents for teachers and residents were presented.

The eligibility criteria for this study included: (1) psychiatry residents who had participated in at least one digital Metis course and (2) course directors/teachers who had taught in at least one digital Metis course during the COVID-19 pandemic. To recruit residents, an email was sent out to all users of the learning management system (via Ping-Pong) used for all Metis courses. Study information was also posted on social media. Study information was also sent out to the principal administrators at the regional councils who in turn forwarded the information to eligible course directors/teachers within their regional networks.

Data collection was ongoing between May-August 2021. An online survey was sent out to 725 residents and 237 course directors/teachers (Table 1) to assess their perceptions of digital psychiatry courses. Data were collected online and managed via an electronic data capture tool hosted in Sweden [5, 6].

**Table 1:** Demographic characteristics of study participants

	Group		Number	Percent
**Invited to participate**	**Teacher**		237	–
	**Resident**		725	–
**Responded**	**Teacher**		79	33.3
	**Resident**		119	16.4
**Fully completed questionnaire**	**Teacher**		72	30.4
	**Resident**		112	15.4
**Gender**	**Teacher**		72	
		Male	29	40.3
		Female	43	59.7
	**Resident**		112	
		Male	38	33.9
		Female	72	64.3
		Unknown	2	1.8
**Years of residency**	**Resident**		112	
		First	11	9.8
		Second	23	20.5
		Third	34	30.4
		Fourth	19	17.0
		Fifth or later	25	22.3
**Healthcare regions in Sweden**	**Resident**		112	
		Northern	9	8
		Central	21	18.8
		Stockholm area	27	24.1
		South East	14	12.5
		South	13	11.6
		West	28	25
**Teaching in digital Metis courses**	**Teacher**		72	
		One	50	69.4
		Two	18	25.0
		Three or more	4	5.6
**Participation in digital Metis courses**	**Resident**		112	
		One	24	21.4
		Two	31	27.7
		Three or more	57	50.9
**Received a walkthrough of digital tools**	**Teacher**			
		Yes	39	54.2
		No	33	45.8

The development of the attitudes survey was partially inspired by the seven-step process of developing questionnaires for educational research [7]. Members of the research group with expertise in the Metis model developed a draft of items. An educational developer helped fine-tune the items and response scales. The survey was then pilot-tested by members from the two target groups and further revised to clarify some statements. The full survey included 53 items for residents and 45 items for course directors/teachers. The present study reports the majority of data obtained, but not all items. The questions were constructed as a 5-point Likert scale, with some open questions (results not presented in this study). The survey is available from the corresponding author on request.

Likert responses for statements such as “The use of interactive tools had a positive effect on the participant’s learning” were assigned the following scores ranging between 1 “Do not agree at all” to 5 “Agree completely”; the “Don’t know” option was interpreted as “Neither Agree nor Disagree” and given a score of 3. Two items “Do you think that Metis course meetings should continue in digital format post-pandemic?” and “What factors influence which course format you prefer?”, were coded (response choices) as 0 or 1, where each statement checked was assigned a value of 1. Data were analysed using SPSS Statistics for Windows, version 26 [8] and JASP statistical software for analysis, version 0.16.2 [9].

Descriptive statistics were used to examine the characteristics of the study population, and independent samples, *t*-tests, to examine differences between study groups. Levene’s test was used to assess the homogeneity of variance for each *t*-test; consequently, when the assumption of homogeneity of variance was not met, the SPSS output for equal variances not assumed was used. A Pearson correlation coefficient was computed to assess the linear relationship between age and perceptions of digital formats. An Analysis of Covariance (ANCOVA) was performed using the General Linear Modeling approach in JASP on response scores for the question “Interactive tools were used in the teaching” as a dependent variable to evaluate the effect of age and of receiving a walkthrough of digital tools before teaching.

### Ethics approval and consent to participate

The need for ethic approval was waived by the The Swedish Ethical Review Authority (Advisory statement; ID# 2021–01920). All participants provided informed consent electronically for participation and to complete the questionnaire. All methods were carried out in accordance with relevant guidelines and regulations.

## Results

The age of residents (*n* = 112) and directors/teachers (*n* = 72) who fully completed the survey (Table 1) ranged between 25–65 years (*M* = 37.39, *SD* = 6.71) and 31–72 years (*M* = 52.56, *SD* = 10.87), respectively.

Perceptions of digital formats during the COVID-19 pandemic were quite similar between the two study groups with some significant differences (Table 2). As illustrated in Tables 2, 41.7% of course directors/teachers and 46.4% of residents preferred a return to the original format with classroom-based course meetings.

**Table 2:** Differences between psychiatry residents and course directors/teachers in perceptions of digital teaching formats during the COVID-19 pandemic and their preferences regarding learning formats for future Metis-courses

										95% Confidence Interval of the Difference	
Item	Group	N	%	Mean	SD	SE	Mean Difference	***P***	SE Difference	Lower	Upper
My digital skills felt adequate	Teacher	72	–	3.93	0.92	0.11					
	Resident	112	–	4.73	0.52	0.05	−0.80	**0.00**	0.12	−1.04	−0.57
My motivation to attend/teach a Metis course was negatively affected by the fact that the course meeting days were digital	Teacher	72	–	2.31	1.32	0.16					
	Resident	112	–	2.51	1.57	0.15	−0.20	0.34	0.21	− 0.63	0.22
Digital Metis courses overall have been of the same quality (or better) than Metis courses in physical format	Teacher	72	–	2.60	1.18	0.14					
	Resident	112	–	3.13	1.30	0.12	−0.53	**0.01**	0.19	− 0.90	−0.15
Interactive tools were used in the teaching	Teacher	72	–	2.60	1.50	0.18					
	Resident	112	–	3.46	1.33	0.13	−0.87	**0.00**	0.22	−1.30	− 0.44
The use of interactive tools had a positive effect on learning	Teacher	72	–	3.00	1.20	0.14					
	Resident	112	–	3.52	1.27	0.12	−0.52	**0.01**	0.19	− 0.89	− 0.15
The participants could collaborate and discuss with each other in a digital format	Teacher	72	–	3.43	1.34	0.16					
	Resident	112	–	3.15	1.34	0.13	0.28	0.17	0.20	−0.12	0.68
Technical problems had a negative effect on teaching/learning	Teacher	72	–	1.92	1.00	0.12					
	Resident	112	–	2.12	1.20	0.11	−0.20	0.24	0.17	− 0.54	0.14
Do you think that Metis course meetings should continue in digital format post-pandemic?*											
Yes, continue with course meetings in a completely digital format	Teacher	72	8.3	0.08	0.28	0.03					
	Resident	112	23.2	0.23	0.42	0.04	−0.15	**0.01**	0.05	−0.25	−0.05
Yes in part, by replacing parts of the classroom-based course meeting days with digital elements	Teacher	72	40.3	0.40	0.49	0.06					
	Resident	112	23.2	0.23	0.42	0.04	0.17	**0.02**	0.07	0.03	0.31
I have no opinion	Teacher	72	5.6	0.06	0.23	0.03					
	Resident	112	0.9	0.01	0.09	0.01	0.05	0.11	0.03	−0.01	0.10
Maybe, it depends on how the digital format develops	Teacher	72	23.6	0.24	0.43	0.05					
	Resident	112	22.3	0.22	0.42	0.04	0.01	0.84	0.06	−0.11	0.14
No, return to the original format with classroom-based course meetings	Teacher	72	41.7	0.42	0.50	0.06					
	Resident	112	46.4	0.46	0.50	0.05	−0.05	0.53	0.08	−0.20	0.10
What factors influence which course format you prefer?*											
My choice depends on the geographical location of the classroom-based course	Teacher	72	40.3	0.40	0.49	0.06					
	Resident	112	55.4	0.54	0.50	0.05	−0.14	0.06	0.08	−0.29	0.01
My choice depends on whether we are in an ongoing pandemic with travel restrictions	Teacher	72	72.2	0.72	0.45	0.05					
	Resident	112	57.1	0.57	0.50	0.05	0.15	**0.04**	0.07	0.01	0.29

*Multiple responses were possible

There was a significant effect of age with regard to preferring course meetings to continue in a digital format post-pandemic; responders < 50 years were more positive (*M* = 0.21, *SD* = 0.41) than responders > 50 years (*M* = 0.08, *SD* = 0.28), *t*(125) = − 2.38, *p* = 0.02.

A negative correlation between age and preference to use interactive tools among course directors/teachers was found *r*(70) = − 0.30, *p* < 0.05. There was a significant effect of receiving a walkthrough of digital tools before teaching on the use of interactive tools in teaching after controlling for age, F(1,69) = 11.47, *p* = 0.001, ω2 = 0.118. Post hoc testing revealed that receiving a walkthrough of digital tools before teaching resulted in a significantly greater use of interactive tools compared to no walkthrough group (*p* < 0.01).

There were gender differences for some items; males displayed a higher preference (*M* = 0.60, *SD* = 0.49) to return to the original format with classroom-based course meetings compared to females (*M* = 0.37, *SD* = 0.48), *t*(180) = 3.09, *p* < 0.01. On the other hand, females (*M* = 3.23, *SD* = 1.21) were more inclined than males (M *=* 2.66, *SD* = 1.11) to think that the use of interactive tools had a positive effect on learning, *t*(70) = − 2.05, *p* < 0.05. Finally, for females, the preferred course format was influenced by the geographical location of the classroom-based course (*M =* 0.54, *SD* = 0.50) to a higher degree than for males (*M* = 0.39, *SD* = 0.49), *t*(180) = − 1.98, *p* = 0.05.

As explained in more detail in the Methods section, Metis courses consist of three phases (i.e., a distance-based Phase I, a classroom-based Phase II, and a distance-based Phase III). Part of the survey explored residents’ and teachers’ attitudes regarding the use of digital elements in different course phases. In both groups, approximately 60% thought Phase I would be strengthened by more digital elements with the following figures for residents and course directors/teachers respectively: video-based lectures (73.2 and 68.1%), sound-recorded lectures (47.3 and 18.1%), digital-group discussions (33.9 and 51.4%), or virtual-patients (48.2 and 45.8%). There was a significant difference in attitudes towards the potential benefits of digital-group discussion; teachers (*M* = 0.51, *SD* = 0.50) believed to a higher degree than residents (*M* = 0.34, *SD* = 0.48) that having a digital forum for group discussions and feedback would benefit the courses in Phase I, *t*(145) = 2.35, *p* < 0.05. On the other hand, residents (*M* = 0.47, *SD* = 0.50) saw more potential in sound-recorded lectures (e.g., podcast format) than teachers (*M* = 0.18, *SD* = 0.39), *t*(176) = − 4.45, *p* < 0.001. For Phase II, the two options with the highest rates in both groups were digital-group discussions (44.6 and 52.8%) and virtual patients (51.8 and 59.7%), for residents and course directors/teachers, respectively. In Phase III, the two options most highly rated were digital-group discussions (40.2 and 52.8%), and virtual patients (50.9 and 51.4%), for residents and course directors/teachers, respectively.

## Discussion

To our knowledge, this is the largest survey study in Sweden, focusing on residents’ and teachers’ perceptions of the digital format of nationwide didactic courses for psychiatry residents. The results demonstrate that participants in both groups seem to have appreciated several aspects of the digital format, even though findings point towards a preference for a “blended course format” in the future. Residents were more positive about the quality of digital courses than study directors/teachers. However, residents were slightly less inclined than course directors/teachers to think that the digital format had facilitated collaboration and discussions. A survey of teachers from Stockholm University (where data collection took place during the same period of the pandemic as the current study) demonstrates that students participated more actively in discussions when the course was on campus compared to online [10]. Participants in this study were undergraduate students (i.e., in contrast to graduate residents in our study), however, the similar trends in the results are worth noting.

In our study, more than 40% of both groups thought that the courses should return to the original format with classroom-based course meetings, also in line with findings by Heldt et al. [3]. Residents, however, to a larger extent were inclined to prefer continuing with course meetings in a completely digital format, whereas teachers showed a preference for replacing some parts of the classroom-based course meetings with digital elements. These mixed reflections are in line with findings from a survey conducted with teachers and students at Stockholm University [10, 11]. The results of that study indicated that even though a majority of course directors/teachers reported feeling comfortable teaching online, they preferred to not teach online in the future, even though they were open to mixing digital and physical formats. Students, on the other hand, were to a larger degree positive about continuing with online courses. Prerequisites for skills and interactive pedagogical coursework may have influenced and increased the quality of the courses to a large extent. In our study, a walkthrough of digital tools influenced teachers’ use of such tools. The result may largely reflect confidence and skill training to teach at a distance after faculty training, as also reported in the study by Heldt et al. [3].

In relation to sex differences, the current study found that there was a preference among males’ for returning to the original classroom-based course format and these findings are also consistent with findings by Bolander Laksov et al. [11], showing that male students preferred physical course meetings to online courses. A study from Malaysia demonstrated that female students on the other hand preferred online learning during the COVID-19 pandemic [12]. Our data showed that females were more inclined than men to perceive interactive tools as positive for learning and that their choice of course formats depended on geographical proximity. All these different aspects above indicate a need to further explore why perceptions of digital formats differ between groups and gender, as well as which digital aspects can be successfully incorporated into classroom-based courses.

Participants in this study were asked to specify which digital elements they thought would be worth incorporating into the different phases. In both groups, participants suggested an elaboration of digital elements in Phase I. This may be due to a lack of variation about assignments in Phase I, possibly indicating that residents do not find these types of assignments stimulating enough. Also, Phase I lacks coherence with the other two phases. The new generation of residents may be more inclined to obtain knowledge through interaction with others, such as via digital synchronous/asynchronous group discussions or by visual or auditory format vs reading only. Strengthening the preparatory phase, i.e., with video-based lectures may contribute to more thorough preparation and allow more time for discussions during Phase II. Participants in both groups suggested the inclusion of digital-group discussions in Phases II and III, indicating that they see some value in digital interactions, even though other results imply that the digital format of Metis courses somewhat reduced networking abilities.

A relatively large degree of participants suggested that virtual patients or “similar digital forums where one can practice the application of central theoretical knowledge”, could be a valuable addition to all three course phases. Indeed, virtual patients and patient simulation could constitute an innovative form of digital teaching tool that could be applied in medical education, specifically within psychiatry [13, 14]. A recently published systematic review regarding digital learning in medical education in the context of the COVID-19 pandemic demonstrated that students reported difficulties in clinical skills acquisition in online learning [15]. Altogether, it may suggest that more focus in curriculum development should address the use of digital elements, such as virtual patients and patient simulations to provide possibilities to acquire clinical skills in the framework of resident courses in digital formats.

The present study assessed attitudes one year after the outbreak of the COVID-19 pandemic in Sweden. Due to time constraints (i.e., we wanted to examine perceptions of digitalized courses while they were still ongoing), a standardized validity and reliability test of the survey was not carried out. It was pilot tested, however, on a group of residents in psychiatry to ensure that the questions were comprehensible and relevant which could be considered a broader validity test. The survey was carried out during the pandemic and there might be a certain degree of bias in the sample, given that there is no control group or baseline evaluation data before the pandemic to compare with. Respondents might be in favour of digitalization overall (i.e., residents opposed to digitalized formats might have skipped out on participating). Another limitation is the sample size. Even though the response rate for residents (15.4%) was low, the corresponding figure for teachers (30.4%) was higher so the overall response rate could be considered reasonable. Both study groups can be considered representative within the national Metis network (e.g., based on age range, course experience, and residents’ geographical spread). To ensure the anonymity of teachers/course directors, their geographical location was not assessed. The “exposure to Metis courses” differed between groups; whereas most residents participated in several Metis courses during the study period, most teachers only taught one course. This discrepancy might have influenced attitudes towards the digital format (e.g., led to a preference among residents for the digital modality to save the time of travelling). Finally, in this study, we assessed perceptions regarding digital formats for Metis courses overall (i.e., it was not possible to break down the results based on any specific course concept).

## Conclusion

The results point towards combining in-person teaching and digital formats. However, striving towards a mixed format would require a plan for curriculum development with continuous evaluations to access which formats are efficient and what purposes they serve [2, 3, 16]. For a revised curriculum to be successfully implemented, the developmental work should include several “checkpoints” [2, 16, 17]. In the Metis context, this could encompass the creation of a few pilot courses (i.e., with new digital elements in Phase I/II or III) along with their evaluation which would involve several steps for residents, teachers/course directors as well as the Metis national network and Board of Directors.

## Data Availability

The datasets analysed during the current study are available from the corresponding author upon reasonable request.

## References

[1] Schwartz AC, Brenner AM (2021). Psychiatric education and COVID-19: challenges, responses, and future directions. Acad Psychiatry.

[2] Knez R (2022). The pedagogical challenges of teaching psychiatry residents during the COVID-19 pandemic: applications for future educational activities. Medicina Flum.

[3] Heldt JP, Agrawal A, Loeb R, Richards MC, Castillo EG, DeBonis K (2021). We’re not sure we like it but we still want more: trainee and faculty perceptions of remote learning during the COVID-19 pandemic. Acad Psychiatry.

[4] Koraym H, Kaltman S, Mete M, Akil M (2021). Remote learning in psychiatry residency programs during COVID-19: emergency measure or path for the future?. Acad Psychiatry.

[5] Harris PA, Taylor R, Thielke R, Payne J, Gonzalez N, Conde JG (2009). Research electronic data capture (REDCap)—a metadata-driven methodology and workflow process for providing translational research informatics support. J Biomed Inform.

[6] Harris PA, Taylor R, Minor BL, Elliott V, Fernandez M, O’Neal L (2019). The REDCap consortium: building an international community of software platform partners. J Biomed Inform.

[7] Artino AR, la Rochelle JS, Dezee KJ, Gehlbach H (2014). Developing questionnaires for educational research: AMEE guide no. 87. Med Teach.

[8] Corp IBM (2019). IBM SPSS statistics for windows, version 26.0.

[9] JASP Team. JASP, version 0.16.2 [computer software]. Amsterdam: University of Amsterdam; 2022. [cited 2022 May 27]. Available from: https://jasp-stats.org/.

[10] Bolander Laksov K, Ismayilova K. Responses to emergency remote teaching at Stockholm University - the teacher perspective [Internet]. Stockholm: Stockholm University; 2021. Reports on Teaching and Learning in Higher Education; No. 2021:4. [cited 2022 Feb 25]. Available from: 10.17045/sthlmuni.15172737.v1.

[11] Bolander Laksov K, Ismayilova K, Curtis R. Responses to emergency remote teaching at Stockholm University - the student perspective [Internet]. Stockholm: Stockholm University; 2021. Reports on Teaching and Learning in Higher Education; No. 2021:3. [cited 2022 Feb 25]. Available from: 10.17045/sthlmuni.14564145.v1.

[12] Chung E, Subramaniam G, Dass LC (2020). Online learning readiness among university students in Malaysia amidst COVID-19.&nbsp;. Asian Journal of University Education..

[13] Martin A, Weller I, Amsalem D, Adigun A, Jaarsma D, Duvivier R (2021). From learning psychiatry to becoming psychiatrists: a qualitative study of co-constructive patient simulation. Frontiers in Psychiatry.

[14] Dupuy L, de Sevin E, Cassoudesalle H, Ballot O, Dehail P, Aouizerate B, Cunv E (2021). Guidelines for the design of a virtual patient for psychiatric interview training. J Multimodal User Interfaces.

[15] Naciri A, Radid M, Kharbach A, Chemsi G (2021). E-learning in health professions education during the COVID-19 pandemic: a systematic review. J Educ Eval Health Prof.

[16] Warren N, Parker S, Khoo T, Cabral S, Turner J (2020). Challenges and solutions when developing online interactive psychiatric education. Australas Psychiatry.

[17] Nagendrappa S, de Filippis R, Ramalho R, Ransing R, Orsolini L, Ullah I (2021). Challenges and opportunities of psychiatric training during COVID-19: early career psychiatrists’ perspective across the world. Acad Psychiatry.

